# Effects of exercise intervention on executive function of middle-aged and elderly people: A systematic review of randomized controlled trials

**DOI:** 10.3389/fnagi.2022.960817

**Published:** 2022-08-12

**Authors:** Jian Zheng, Xuan Su, Chang Xu

**Affiliations:** ^1^School of Psychology, Shanghai University of Sport, Shanghai, China; ^2^School of Kinesiology, Shanghai University of Sport, Shanghai, China

**Keywords:** executive function, exercise intervention, elderly people, middle-aged people, system review

## Abstract

**Background:**

Executive function will gradually decline with the increase of age, which will have a negative impact on the quality of life and general health. Exercise intervention can improve executive function and prevent its deterioration, but the evidence from randomized controlled trials is not consistent.

**Aim:**

To assess the effect of exercise intervention on executive function of healthy middle-aged and elderly people, and briefly describe its mechanism.

**Methods:**

A search was conducted using PubMed, Web of science and EBSCO. The searches were limited to English articles published from January 2010 to January 2022. The information is extracted from searched articles included or excluded based on certain criteria.

**Results:**

The search returned 2,746 records, of which 11 articles were included in the systematic review, and 8 articles were supplemented according to the references included and related reviews. The results show that different types of exercise intervention have positive effects on the executive function of the middle-aged and elderly people. The intervention prescriptions in most studies are executed in medium to low frequency, medium intensity and medium duration, while only 11% of the studies were followed up.

**Conclusions:**

The intervention, which was executed twice a week with 30–60 min single intervention time and over 12 weeks total duration, showed a good intervention effect. Exercise intervention is to improve executive function by affecting the activation of brain network and the synthesis of neurotransmitters.

## Introduction

Executive function (EF) is the advanced cognitive ability of individuals, as well as the core of cognitive, emotional and social functions. The core content of EF includes three aspects: inhibition control, working memory and cognitive flexibility (Miyake et al., 2000). The decline of EF will seriously impact people's daily life, as they both closely related to each other. When the EF is impaired, it will be difficult for individuals to focus their attention on a certain point, or they will appear behavioral reactions that deviate from the target, as well as the symptoms of memory persisting declination, etc. (Chang et al., 2014). If there is no timely intervention and prevention, it can lead to inability of coordination of thinking and behavior to adapt to the changes of the environment, then gradually developing into Alzheimer's disease (AD), which has a great impact on the quality of life of the middle-aged and elderly people. Therefore, improving the EF of the middle-aged and elderly people is of great significance in improving the quality of life in the late stage of life and realizing the successful aging of the elderly people.

Distinguished from drug therapy, exercise intervention is a way to improve EF, which has attracted the attention of researchers *via* its safety and non-invasive. It has been proved and reported by a large number of studies that the appropriate exercise intervention or physical activity could bring beneficial effect to human health. Spirduso was the first to find a positive effect of physical activity on cognitive function (Spirduso and Clifford, 1978). Epidemiological studies have shown that people who take moderate exercise have a lower risk of mental disorders than sedentary people. And the beneficial effects of physical activities are most obvious in the EF (Etnier and Chang, 2009).

A randomized controlled trial (RCT) is a type of scientific experiment that aims to reduce certain sources of bias when testing the effectiveness of new treatments. Results of RCTs may be combined in systematic reviews which are increasingly being used in the conduct of evidence-based practice (Chalmers et al., 1981). The results from RCTs show that different forms of exercise including aerobic exercise, resistance exercise and physical and mental exercise for a period of time can promote memory updating, inhibition control and other EF of healthy middle-aged and elderly people (Northey et al., 2018). Also, the appropriate level of physical activities can help the elderly people with mild cognitive impairment to improve the EF to a certain extent (Zheng et al., 2016), even effectively prevent and delay the occurrence and development of AD (Cammisuli et al., 2017; Panza et al., 2018b).

However, other RCTs have pointed out that exercise intervention cannot improve cognitive and EF, high-intensity exercise may cause exercise fatigue even damage to EF (Van Duinen et al., 2007). The inconsistent results observed in RCTs make it impossible for clinical workers and researchers to summarize more accurately on the effects of exercise intervention and cognitive health of the middle-aged and elderly people. In addition, there are some experimental design defects and method limitations in the previous system review. In addition, although some researchers have made relevant reviews in the field of exercise affecting executive function, there are few reviews on middle-aged and elderly people. And few researchers focus on exercise prescription variables to explore and review the impact of exercise on executive function. Thus, it is necessary to systematically review the newly published RCTs on the effects of exercise intervention on the EF of the middle-aged and elderly people.

Therefore, this systematic review of RCTs attempts to clarify the differences observed in recently published RCTs. It also discusses the influence of exercise prescription variables on EF and the mechanism of exercise intervention to improve the EF of the middle-aged and elderly people, so as to provide reference for formulating intervention strategies and improving the quality of life of the middle-aged and elderly people.

## Methods

### Eligibility criteria

This systematic review included studies that met the following criteria: (a) the subjects were healthy middle-aged and elderly people over 40 years old with normal cognitive function. (b) Intervened target should be the EF (inhibitory, updating and switching) of the middle-aged and elderly people. (c) The experimental method must be a randomized controlled trial. (d) The EF was evaluated after the intervention. (e) Non-middle-aged and elderly people (infants, children, adolescents and others) and non-exercise intervention studies were excluded according to the title of the retrieved literature. (f) Reviews, degree papers, conference papers and non-English articles were not included in the search scope. (g) Studies that did not report detailed data results in full text or abstracts were also excluded.

### Literature search

Systematic searches of the literature published from January 1, 2010 to January 1, 2022 were conducted based on Preferred Reporting Items for Systematic Reviews and Meta-Analyses (PRISMA) guidelines (Moher et al., 2009) using the electronic databases Web of Science, PubMed, EBSCO and MEDLINE The databases were searched by either title or title and abstract. The search items included 4 groups: (a) population: elderly, aging, middle-aged, older. (b) EF: executive function, executive control, inhibition, updating, working memory, switching. (c) Exercise: physical activity, sport, exercise. (d) Experimental design: randomized controlled trials, randomized clinical trials, controlled clinical trials.

### Study selection

First of all, Boolean logic word “AND” and “OR” were to connect the search items for retrieval. Secondly, the full text and references that meet the retrieval conditions were searched manually to supplement the missed documents in the first round of retrieval. Finally, the references of the reviewed literature were searched manually to supplement the missing documents in the first two rounds of retrieval.

### Data extraction

The literature information is extracted by the two authors, respectively, and the inconsistent information is judged by the communication between the two authors. The data extracted from the final included literature includes: first author, year of publication, sample characteristics, exercise intervention prescription, execution of functional tasks, and result measurement. The specific literature information included in this review is shown in Tables 1, 2, established on the basis of Table 1, is the result of a quantitative summary of the characteristics of each study.

**Table 1:** Intervention characteristics of included studies.

**Study**	**Grouping**	**Exercise characteristics**	**Sample characteristics**	**CF or EF Task**	**Outcome measurement**
Martin-Willett et al. (2021)	LICT group (*n* = 64) MICT+IT group (*n* = 78)	Freq.: 3 days/week Int.: 50% HRmax (LICT) and 60-95% HRmax (MICT+IT) Type: AE Time: 30 min Length: 16 weeks	Age: >60 Gender: Both PF: Sedentary	Stroop Keep Track task Category Switch task	Compared with the baseline, both: EF↑^*^
Kleinloog et al. (2019)	Intervention group (*n* = 17) Control group (*n* = 19)	Freq.: 3 days/week Int.: 70% Pmax Type: AE Time: 50 min Length: 32 weeks	Age: >65 Gender: Male PF: Sedentary	MTT SSP	Compared to the control group, Intervention: frontal lobe CBF↑^*^, OGTT↓^*^, EF↑^*^
Norouzi et al. (2019)	mMtt group (*n* = 20) mCtt group (*n* = 20) Control group (*n* = 20)	Freq.: 3 days/week Int.: NM Type: RE Time: 60–80 min Length: 4 weeks	Age: 60–70 Gender: Male PF: Fit	n-back	Compared to the control group, The other 2: EF (WM)↑^*^; mCtt group improved more significantly
Wang et al. (2018)	Experimental group (*n* = 16) Control group (*n* = 11)	Freq.: 3 days/week Int.: 70–75% HRmax (ET) and 75–80% (RE) Type: Multicomponent exercise Time: 60 min Length: 12 weeks	Age: >65 Gender: Both PF: Fit	C-EXIT25	Compared to the control group, Experimental: EF↑^**^, GP↑^**^
Tsai et al. (2017)	Closed-skill group (*n* = 22) Open-skill group (*n* = 21) Control group (*n* = 21)	Freq.: 3 days/week Int.: 70–75% HRmax (AE) NM (CE) Type: AE and CE Time: 30 min (AE) 40 min (CE) Length: 24 weeks	Age: 60–80 Gender: male PF: Sedentary	N-back Task switching	Compared to the control group, The other 2: TS RT↓^*^, n-back ACC↑^*^, P3 amplitudes↑
Albinet et al. (2016)	Swimming (*n* = 19) Stretching (*n* = 17)	Freq.: 2 days/week Int.: 40–65% HRR Type: AE Time: 40 min Length: 21 weeks	Age: 60–75 Gender: Both PF: Sedentary	Stroop RNG Hayling Task RST N-back DST Plus-Minus task DLT	Compared to the stretching group, Swimming: HRV↑^*^, Inhibition↑^*^, WM^*^↑,
Falbo et al. (2016)	Single task group (*n* = 16) Physical-cognitive dual task group (*n* = 20)	Freq.: 2 days/week Int.: NM Type: CE Time: 60 min Length: 12 weeks	Age: 65–80 Gender: Both PF: Fit	RNG task	Compared with the baseline, both: EF↑^*^; Physical-cognitive dual task group improved more significantly
Eggenberger et al. (2015)	VR dance group (*n* = 24) Memory group (*n* = 22) Walking group (*n* = 25)	Freq.: 2 days/week Int.: NM Type: AE Time: 60 min Length: 24 weeks	Age: >70 Gender: Both PF: Fit	TMT-B ECT	Compared to the VR dance group, The other 2: EF↑^*^; Memory group improved more significantly
Iuliano et al. (2015)	Resistance group (*n* = 20) Cardiovascular group (*n* = 20) Postural group (*n* = 20) Control group (*n* = 20)	Freq.: 3 days/week Int.: 80–85% 1 RM (RE) 70–80% HRR (AE) Type: RE and AE Time: 30 min Length: 12 weeks	Age: >55 Gender: Both PF: Sedentary	Stroop TMT	Compared to the control group, The other 3 groups: Stroop RT and ACC=, TMT=
Barcelos et al. (2015)	Tour group (*n* = 34) Game group (*n* = 30)	Freq.: 3–5 days/week Int.: 80% HRmax Type: ACE Time: 20–45 min Length: 12 weeks	Age: 82.2 Gender: Both PF: Fit	Stroop test DST Color Trails 1 and 2	Compared with the baseline, both: EF↑^*^; Game group improved more significantly
Gothe et al. (2014)	Yoga group (*n* = 61) Control group (*n* = 57)	Freq.: 3 days/week Int.: NM Type: Yoga Time: 60 min Length: 8 weeks	Age: 55–79 Gender: Both PF: Sedentary	Task switching running memory span N-back	Compared to the control group, Yoga: WM↑^*^, Shifting↑^*^
Vaughan et al. (2014)	Intervention group (*n* = 25) Control group (*n* = 23)	Freq.: 2 days/week Int.: NM Type: Combined exercise Time: 60 min Length: 16 weeks	Age: 65–75 Gender: Female PF: Fit	TMT LNS Stroop Test COWAT	Compared to the control group, intervention: WM↑^*^, BDNF↑^*^
Nouchi et al. (2014)	Combination group (*n* = 32) Control group (*n* = 32)	Freq.: 3 days/week Int.: 60–80% HRmax Type: Combined exercise Time: 30 min Length: 4 weeks	Age: >60 Gender: Both PF: Sedentary	VFT DST Stroop test	Compared to the control group, Combination: EF↑^*^
Fallah et al. (2013)	RT group 1 (*n* = 52) RT group 2 (*n* = 54) BAT group (*n* = 49)	Freq.: 1–2 days/week Int.: 7 RM Type: RE Time: 60 min Length: 52 weeks	Age: 65–75 Gender: Female PF: Fit	Stroop test TMT VDT	Compared to the BAT group, RT 1 and 2: EF↑
Dao et al. (2013)	RT group 1 (*n* = 37) RT group 2 (*n* = 41) BAT group (*n* = 36)	Freq.: 1–2 days/week Int.: NM Type: RE Time: 60 min Length: 52 weeks	Age: 65–75 Gender: Female PF: Fit	Stroop test	Compared to the BAT group, RT 1 and 2: inhibition↑^*^
Maillot et al. (2012)	Training group (*n* = 16) Control group (*n* = 16)	Freq.: 2 days/week Int.: NM Type: NM Time: 90 min Length: 14 weeks	Age: 65–78 Gender: Both PF: Fit	Stroop test TMT LST	Compared to the control group, Training: inhibition↑^*^, WM↑^*^
Klusmann et al. (2010)	Exercise group (*n* = 91) Control group (*n* = 76)	Freq.: 3 days/week Int.: NM Type: Combined exercise Time: 90 min Length: 24 weeks	Age: >70 Gender: Female PF: Fit	VFT Stroop Test TMT	Compared to the control group, Exercise: inhibition↑^*^, WM↑^*^
Liu-Ambrose et al. (2010)	RT group 1 (*n* = 52) RT group 2 (*n* = 54) BAT group (*n* = 49)	Freq.: 1–2 days/week Int.: 6–8 RM Type: RE Time: 60 min Length: 48 weeks	Age: 65–75 Gender: Female PF: Fit	Stroop test TMT VDS	Compared to the BAT group, RT 1 and 2: inhibition↑^*^, WM↑
Kimura et al. (2010)	Exercise intervention group (*n* = 86) Health education group (*n* = 85)	Freq.: 2 days/week Int.: 60% 1RM Type: RE Time: 90 min Length: 12 weeks	Age: >65 Gender: Female PF: Fit	Task switching	Compared to the health education group, Intervention: HRQOL↑, EF=

*Freq., frequency, Int., intensity; PF, physical fitness level; CF, cognitive function; MTT, the multitasking test; SSP, spatial span; RE, resistance exercise; AE, aerobic exercise; ET, endurance training; mMtt, a motor-motor dual-task training; mCtt, a motor-cognitive dual task training; C-EXIT25, The Chinese version 25-item executive interview; CE, coordination exercise; NM, not mentioned; RNG, the random number generation; RST, running span task; DST, the dimension-switching task; DLT, the digit-letter task; TMT, trail making test; ECT, executive control task; RM, repetition maximum; HRR, heart rate reserve; LNS, the letter-number sequencing; COWAT, control oral word association test; VFT, verbal fluency test; VDT, verbal digits forward and backward tests; BAT, balance and tone; VDS, verbal digit span; ACE, aerobic and cognitive exercise; CBF, cerebral blood flow; OGTT, oral glucose tolerance test; GP, gait performance; TS, task switching; HRV, cardiac vagal control; WM, working memory; BDNF, brain derived neurotrophic factor; HRQOL, health-related quality of life; =, No change or no difference; ↑, Elevated or higher;*

* *p < 0.05;*

** *p < 0.01*.

**Table 2:** Summary of intervention characteristics of included studies.

**Characteristics**	**Percentage (%)**	**Characteristics**	**Percentage (%)**	**Characteristics**	**Percentage (%)**
**Published year**		**Time**		Both	52.6
2016–2022	36.84	<30 min	5.3	**PF**	
2010–2015	63.16	30–60 min	68.4	Fit	68.4
**Exercise type**		>60 min	21.1	Sedentary	31.6
Aerobic	26.3	**Sample size**		**EF domains**	
Resistance	26.3	<30	5.3	Inhibition	66.7
Combined	21.0	30–60	36.8	Updating/WM	83.3
Others	26.3	61–90	26.3	Shifting	50.0
**Frequency**		>90	31.6	**Tools**	
<2	15.8	**Age**		Stroop test	55.6
2–4	78.9	55–65	5.3	N-back	22.2
>4	5.3	65–75	84.2	TMT	38.9
**Length**		>75	10.5	Task switching	16.7
<12	15.8	**Gender**		Others	11.1
12–24	57.9	Male	15.8		
>24	15.8	Female	31.6		

*PF, physical fitness level*.

### Risk of bias in individual studies

The quality and level of the evidence of included studies were assessed by two authors using the Cochrane Collaboration's tool for assessing risk of bias for RCTs (Higgins et al., 2011). Each item is judged independently by two authors on the risk of material bias. When there is a difference in the evaluation between the two authors, it must be discussed.

### Subcomponent of executive function

Miyake et al. believed that executive function is not a single-structure system, but a multi-dimensional cognitive structure composed of multiple sub-functions, including inhibition, updating and switching (Miyake et al., 2000). Researchers often use task paradigms such as Stop-signal, Stroop, Flanker, Simon, and Go/Nogo to measure individual inhibitory control ability. Memory refresh is mostly measured by task paradigms such as alphabet (number) activity memory task, tracking task, number refresh task and N-back paradigm. Attention switching is often assessed using the task switching paradigm. Task types include digital part-of-speech switching, more-odd switching, plus-minus switching, local-global tasks, etc.

## Results

**Figure 1:**
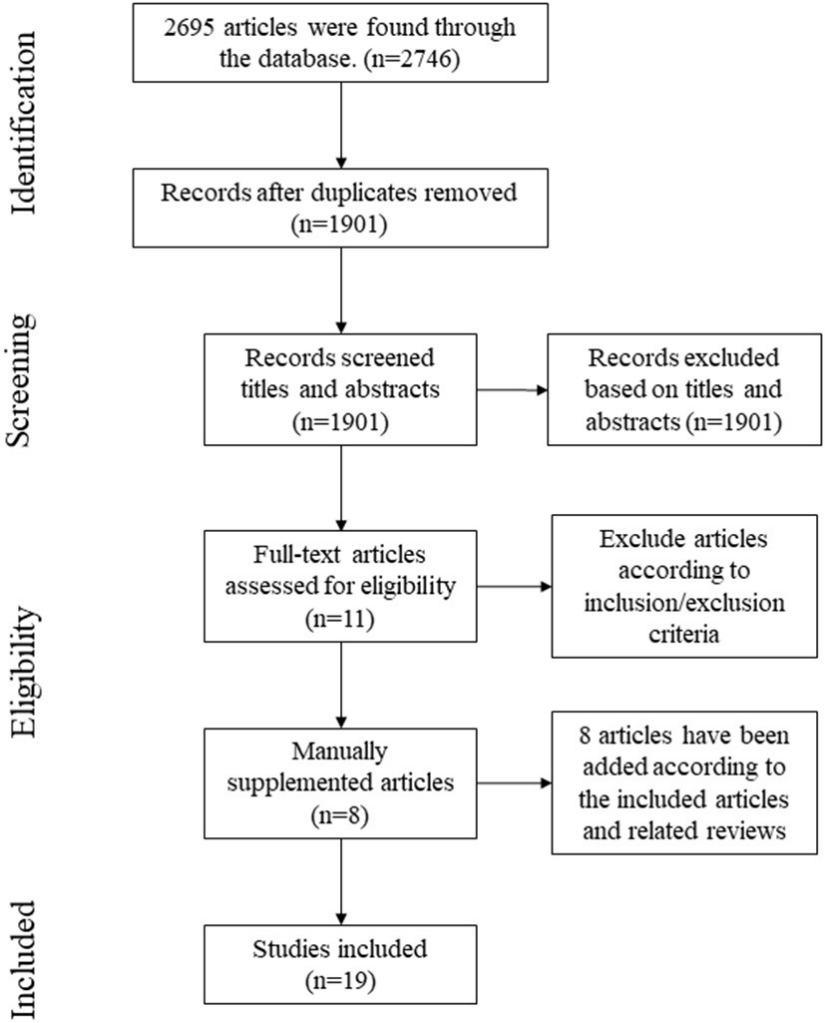
Literature search flow diagram.

As shown in Figure 1, according to the search strategy, a total of 2,746 articles were retrieved, of which, 11 articles were included after screening, and 8 articles were supplemented based on the included references and related reviews. Finally, 19 articles were included with 63.16% articles published from 2010 to 2015 and 36.84% from 2016 to 2022.

### Demographic factors

We found that the average age of 84.2% of the subjects was between 65 and 75 years old. Fifty-five to sixty-five year-old subjects accounted for 5.3% of the total included in the study, and 10.5% were older than 75 years old. In addition, there were no middle-aged subjects under the age of 55 in the included study. 31.6% of the subjects in these studies were sedentary.

In terms of gender, 15.8% of the studies conducted exercise intervention only on men, 31.6% of the studies only on women, and the remaining 52.6% of the studies used overall (male and female) samples for exercise intervention.

### Risk of bias

All the studies included were RCTs designed and reported randomized allocation. All randomized controlled intervention studies used EF assessment tools with good validity. Of these, <10% of the studies used a double-blind design, and 44.4% of the studies used a single-blind design.

The study included with a sample size of 30–60 and more than 90 accounted for 36.8 and 31.6%, respectively. Studies with a sample size of 61–90 accounted for 26.3%. 5.3% of the studies had a sample size of <30 people.

### Intervention prescription

According to Table 1, we can find that so far, various types of exercise intervention measures have been carried out among the middle-aged and elderly people with normal cognitive function, with different intervention duration and frequency.

In terms of the time of single intervention, 68.4% of the research intervention time was concentrated in 30–60 min. 21.1% of the studies had an intervention time of more than 60 min, and another 5.3% of the studies had an intervention time of <30 min. In terms of the frequency of intervention, 78.9% of the studies had an intervention frequency of 2–4 times a week, 15.8% of the studies were <2 times a week, and 5.3% of the studies were more than 4 times a week. From the perspective of the overall intervention cycle, the time of intervention ranges from 4 to 52 weeks. 57.9% of the research intervention time was concentrated in 12–24 weeks, among which, intervention time of 15.8% of the studies was <12 weeks, and 15.8% was more than 24 weeks.

The intervention measures mainly include aerobic training, resistance training, multi-component training and other training. Among them, 26.3% of the studies used aerobic training for intervention studies, while 26.3% used resistance training. In addition, joint training studies accounted for 21.0% of the total included studies, and 26.3% of the studies adopted other training methods such as yoga and Pilates, or specific types of interventions were not mentioned in the text.

Surprisingly, 1/3 of the studies did not mention the specific intensity of exercise intervention. In the studies with recorded intervention intensity, the data were mostly concentrated between 60 and 80%HRmax or 6–8 RM.

## Discussion

Other subcomponents of executive function, such as switching, was seldom measured in previous studies. The current system review included all three aspects of executive function. In addition, this review pays more attention to the influence of exercise prescription variables (e.g., type, frequency, intensity and duration of exercise) on executive function in the middle-aged and elderly population, and attempts to explore which exercise prescription has a better effect on the improvement of executive function.

### Demographic variables and risk of bias

Most of the subjects in the included studies were from 65 to 75 years old. There are few studies on middle-aged and elderly people over 75 years old. Erickson's research shows that, compared with children and the elderly, the research on the impact of physical activity on the EF of young and middle-aged groups is lack of relevant systematic review and META analysis (Erickson et al., 2019). In negligible quantity of studies on this group, the results of the study on the effects of medium-and high-intensity exercise on cognition are not consistent. The lack of longitudinal studies on people over 75 years old will lead to a lack of further understanding of the relationship between the effect of exercise intervention and age. Therefore, additional research is needed on several of the groups mentioned above.

Most studies did not describe whether there were gender differences in the intervention results, as only male (female) subjects were selected in some studies. Although some other studies have investigated both male and female subjects, there is no further discussion on the results. In fact, according to a review of gender differences in the improvement of cognition by physical exercise published by Barha et al. (2017), we can learn that among the effects of exercise intervention on EF, gender is an important regulator of cognitive (executive) function, and women can benefit more from exercise than men for EF (Barha et al., 2017). Other studies have also shown that in terms of EF, studies with a higher proportion of female subjects reported with a larger impact, indicating that gender is an important moderator of the effect of exercise on cognition (Colcombe and Kramer, 2003; Barha et al., 2017). When formulating the prescription of exercise intervention, the future research should fully consider the effects of biological, cognitive and sociological factors on the EF for the middle-aged and elderly people of different genders.

As for physical fitness level, we found that some studies used sedentary people as research subjects. However, one study in sedentary people did not find a significant improvement in executive function with exercise intervention (Iuliano et al., 2015). The explanation given by the authors was that the ineffective outcome of this intervention may be due to the short duration of the high-intensity phase of exercise to produce no significant improvement in executive function. This is slightly different from the results of some later studies. In addition, we found that there seems to be no study comparing the effect of exercise on the improvement of executive function in people with different physical activity levels.

In addition, the studies included are all RCTs to ensure the integrity of the result data and present a complete research plan as far as possible, so that it can minimize the distribution bias and balance the prognostic factors (Higgins et al., 2011). The internationally recognized and widely used CONSORT statement provides a standard method for writing randomized controlled trial reports (Altman et al., 2001), the reports based on which are helpful for people to strictly evaluate the experimental design, explain the results, and find out problems difficult to explain or potential biases. However, only 1/3 of the included studies reported the CONSORT statement, while most of the studies did not provide complete information in the CONSORT statement. It is suggested that future RCTs should be strictly designed based on the CONSORT statement in order to reduce the possible deviation of the experiment.

The samples sizes selected in the experiment ranged from 27 to 171. In more than half of the studies, the number of the sample size was between 30 and 90. Theoretically, the size of the sample depends on the main variables, variance and allowable error. In their research about sample size design, Barlett et al. pointed out that under the condition that the effect is 0.8 and the significance level is 0.05, it is appropriate to take a 21 people group as the minimum sample size (Kotrlik and Higgins, 2001). It is suggested that future studies should be based on this theory when determining the sample size of the intervention experiment. In addition, we should comprehensively consider the maneuverability of the experiment, the control of non-sampling error, budget, etc., and strive to achieve the choice of the optimal sample size.

### Effect of the different types of exercise on EF

Included studies have discussed the effects of exercise interventions such as aerobic exercise, resistance exercise, multi-component exercise and physical and mental exercise. In the early years, there were many studies on aerobic exercise, while in recent years, other kinds of exercise began to be concerned by researchers. At present, the research evidence shows that exercise intervention has the greatest benefit to EF and memory (Colcombe and Kramer, 2003; Northey et al., 2018), which depends on the type of training to a certain extent.

Barha et al. found that aerobic exercise has the greatest influence on cognitive EF (Barha et al., 2017). The report from World Health Organization (WHO) also shows that aerobic exercise is related to the improvement of neurocognitive function, especially EF (World Health Organization, 2008; Guiney and Machado, 2013). Kleinloog et al. conducted a comparative study of aerobic exercise intervention and stretching exercise intervention on healthy elderly men over 65 years old, which found that EF improved as the latency of response was reduced by 5% (*P* = 0.034) in aerobic exercise group (Kleinloog et al., 2019). Another study of 71 healthy elderly people also showed that 24-week aerobic exercise intervention played an important role in improving EF. Among them, 47 subjects showed faster attention switching ability (*p* = 0.075) and stronger working memory ability (*p* = 0.051) after intervention (Baker et al., 2010). In addition, swimming was deeply loved by the elderly people because of its characteristics such as mobilizing the body muscle participation, low pressure on joints, low risk of sports injury and so on. A study published in 2016 showed that the participants in the water exercise group significantly improved their vagally-mediated HRV, as well as their performance for the Stroop test and the verbal running-span test at the end of the program. A study focused on the effects of closed sports such as swimming and open sports effect of table tennis on EF, etc. and so on. The results of which showed that the two exercise modes produced varying degrees of neuropsychological benefits on RT of task switching paradigm (i.e., reducing RTs) and ARs of N-back task (i.e., increasing ARs) (Tsai et al., 2017).

Similar to aerobic exercise, resistance exercise may have different effects on cognitive function and affect the performance of specific tasks. We found that resistance training can effectively improve the inhibition ability and working memory of the middle-aged and elderly people. Liu-Ambrose et al. randomly divided 155 elderly women aged 65–75 into a resistance training groups executed, respectively, once a week, twice a week and a control group. The duration of each intervention was 60 min, including warm-up, formal training and relaxation, with stroop task used to evaluate the subjects' selective attention and inhibition ability. The results showed that the 12-month resistance training intervention had a positive effect on the selective attention and inhibition ability of older women (*p* = 0.03). The weekly and twice-weekly training frequency increased the subjects' performance on the stroop task by 12.6 and 10.9%, respectively, while the control group decreased by 0.5% (Liu-Ambrose et al., 2010). A study in 2019 also showed that 4-week resistance exercise significantly improved the working memory ability of healthy men aged 60–70 years old with a significant time × group interaction compared with the control group (*p* < 0.01) (Norouzi et al., 2019).

Colcombe and Smith believe that the combination of aerobic exercise and resistance training is more effective than aerobic exercise alone in improving the performance of attention and working memory tasks (Colcombe and Kramer, 2003; Smith et al., 2010). A Meta-analysis by Patrick et al. also pointed out that multi-component exercise intervention has a stronger effect on the protection and improvement of attention and working memory of the elderly people than aerobic or anaerobic exercise alone. The reason may be that comprehensive exercise intervention is more helpful to reduce the risk factors of cardiovascular and cerebrovascular diseases, so as to alleviate white matter degeneration and brain ischemia and hypoxia (Patrick et al., 2010). Several recent randomized controlled trial for people over 60 years old have also shown that multi-component exercise can improve EF in different dimensions (Nouchi et al., 2014; Vaughan et al., 2014; Wang et al., 2018). Nouchi et al. randomly assigned 64 healthy elderly people to multi-component exercise group and control group. The multi-component exercise group was intervened 3 days a week (aerobic, strength and stretching training) for 4 weeks (a total of 12 exercises), without the intervention in the control group. The results showed that compared with the control group, the intervention group improved EF, episodic memory ability and information processing speed (Nouchi et al., 2014), based on which, new studies have found that compared with simple multi-component exercise, exercise intervention, combined with cognitive activities, can produce greater cognitive benefits (Eggenberger et al., 2015).

In addition, physical and mental exercises represented by Taijiquan, yoga and Pilates have attracted the researchers' attentions *via* their comprehensive characteristics, including aerobic, anaerobic and flexibility training. The evaluation of EF includes Task switching and running memory span and N-back. The results showed that the performance of EF indexes in the yoga intervention group, such as working memory capacity, mental set transformation and flexibility, was significantly better than that in the control group (Gothe et al., 2014). Therefore, physical and mental exercise can play a certain role in protecting the EF of the elderly people.

In conclusion, different ways of exercise intervention play a positive role in improving and maintaining the EF of the middle-aged and elderly people. Future research should not be limited to the influence of a single type of exercise on the EF of the middle-aged and elderly people. The combined exercise intervention should be considered in the future.

### Effect of exercise intensity, duration and frequency on EF

The reason why exercise intervention can improve the EF of the middle-aged and elderly people is that exercise can promote the process of energy metabolism *via* a certain intensity and load. However, we found that many included RCTs did not mention the explicit intensity of intervention. In these studies, as different types of motion have different standards, unified standards are required to be established for definition and measurement of medium intensity.

A 2017 intervention study explored the effects of HIIT and MCT on executive function in healthy elderly people without training experience from the perspective of overall cognitive function. The exercise intensity of the MCT group was set at 70–75% HRR and the HIIT group was set at 90–95% HRR. After a 16-week intervention trial, it was found that the HIIT group showed a significant improvement in the response time of information processing tasks, that is, Stroop neutral (ES = 1.11). In the MCT group, the reaction time of performing cognitive tasks was significantly improved, that is, Stroop inconsistency and interference (ES 1.28, 1.31, respectively) (Coetsee and Terblanche, 2017). It shows that MCT is better than HIIT in improving the executive function of the elderly people, while HIIT is more conducive to the improvement of information processing speed.

The Physical Activity Guidelines for Americans points out that the intensity of intervention can be divided into absolute intensity and relative intensity. Absolute intensity is the amount of energy expended during the activity, without considering a person's cardiorespiratory fitness or aerobic capacity. It is usually expressed in units of metabolic equivalent (MET). The MET level of moderate intensity exercise ranges from 3 to 5.9 Met. Relative intensity is the ratio of effort required to do an activity to an individual's ability. The relative strength can be estimated on a scale of 0 to 10, in which the sitting posture is 0 and the highest effort level is 10. At this level, the moderate intensity of activity is 5 or 6. This may provide a reference standard of intervention intensity for future research (Piercy et al., 2018).

Northey et al. suggested that exercise of any frequency lasting 45–60 min is beneficial to cognitive function (Northey et al., 2018). However, most of the included studies only considered a single factor, intervention frequency or single intervention time or intervention weeks. Overall, the current research still requires a clear time basis for intervention. From the point of view of the effectiveness of the intervention, it was found that the exercise intervention with a single intervention duration of 30–60 min, at least twice a week and a total duration of more than 12 weeks could significantly exert the intervention effect. However, it is worth noting that two of these studies did not report the effectiveness of exercise intervention on EF (Kimura et al., 2010; Iuliano et al., 2015). This maybe because the intervention time of RCTs is slightly shorter than the time required for exercise intervention on the brain, it is difficult to observe the effect of intervention at the behavioral level. In addition, as the recruited subjects are all healthy middle-aged and elderly people, their EF may be at a relatively healthy level. Therefore, it is difficult to determine the improvement of EF during the intervention period of short-term RCTs. Further follow-up research is needed to for conformation.

The follow-up investigation after the intervention is very important. Epidemiological studies have shown that it may take years for exercise to affect brain health (Panza et al., 2018a). However, we found that in the included studies, only two studies have been conducted follow-up research (Eggenberger et al., 2015; Norouzi et al., 2019), among which, the follow-up study of Patrick et al. shows that the effect of exercise intervention can be maintained for at least 1 year. Even in the TMT-B test, from the 6-month test to the follow-up test, the performance of all groups continued to improve, which may reflect the delayed effect of exercise intervention (Eggenberger et al., 2015). The follow-up study of Norouzi et al. also proved the time effect of exercise intervention on improving working memory (Norouzi et al., 2019). Unfortunately, due to financial and human effect reasons, most of the studies have not been followed up and tested. Therefore, it is impossible to evaluate and analyze the follow-up impact of the intervention. In future studies, when conducting the impact of exercise intervention on EF, it is recommended to conduct a follow-up survey within a period of time after the end of the intervention, which is one of the important ways to evaluate the effectiveness of exercise intervention.

### Possible reasons why exercise affects executive function

The researchers have successively proposed the selective improvement hypothesis (Kramer et al., 1999) and the cardiovascular function hypothesis (Etnier et al., 2006) to explain the mechanism, by which exercise intervention affects EF. The researchers pointed out that the frontal lobe is an important material basis of EF, and aerobic exercise can selectively improve the function dependent on the prefrontal lobe by promoting the aerobic fitness of individuals. However, this theory cannot explain the effects of exercise other than aerobic exercise on general cognitive function. Another theory holds that cardiovascular function is the physiological intermediary variable of exercise affecting cognitive EF, which is related to the changes of brain structure and the levels of BOLD and BDNF caused by exercise.

In recent years, the exercise-cognitive intermediary model (Spirduso et al., 2008) and cognitive reserve theory (Stern, 2002) newly proposed by researchers have attracted more attention. Exercise-cognitive intermediary model not only describes the direct impact of exercise on cognitive function, but also fully reflects the three intermediary variables of physical resources, psychological resources and chronic diseases. However, there is no confirmatory research report on this model. Cognitive reserve theory mainly describes the protective effects of psychological resources and exercise on cognitive function. Under the premise of similar neuropathological symptoms, individuals with higher cognitive reserves can show mild cognitive impairment and pathological changes through potential cognitive protection and compensation mechanisms. Exercise intervention can delay the decline of EF in the middle-aged and elderly people by activating individual cognitive reserve (Fratiglioni et al., 2004) Studies based on this theory show that exercise promotes the balance of energy metabolism, stimulates the brain to synthesize BDNF and binds to cholinergic receptor TrkB to activate corresponding signal transduction pathways to regulate the proliferation and differentiation of nerve cells and promote the growth of new neurons and synapses (Nithianantharajah and Hannan, 2009; Gomez-Pinilla et al., 2011). However, some studies have pointed out that serum BDNF levels in patients with MCI and AD are significantly higher than those in normal people, which is irrelevant to the severity of the disease, antidepressants and the use of acetylcholine inhibitors (Angelucci et al., 2010).

Event-related potential (ERP) technique can record the characteristics of brain information processing in a very short time. N2 and P3 are two components that are paid more attention to in the research of EF. N2 has been associated with the detection of response conflict, the mismatch of a stimulus with a mental template, and/or the upregulation of cognitive control during early stages of response inhibition. The amplitude of P3 is sensitive to the amount of attentional resources engaged during task performance, while the latency is generally believed to index the time required to detect and process a target stimulus (Alderman et al., 2016). Hillman et al. found that the latency of P3 in the prefrontal region of the middle and high intensity aerobic exercise group was significantly shorter than that of the low intensity exercise group. The amplitude of P3 wave of the middle and high intensity aerobic exercise group was larger than that of the young control group (Hillman et al., 2008). Other researchers have found that exercise intervention can increase the overall electrophysiological effects from frontal lobe to parietal cortex (that is, increased amplitude of P3) (Tsai et al., 2017).

Although ERP technology has millisecond time accuracy, it is weak in spatial resolution. FMRI technology can accurately locate the degree of brain activity through blood oxygen-dependent horizontal (BOLD), which can make up for the lack of spatial resolution of ERP technology. Studies have found that exercise can change the activation patterns of brain regions, such as prefrontal lobe (dorsolateral and ventrolateral), cingulate gyrus (anterior cingulate gyrus), cerebellum, parietal lobe and striatum (ventral). The three sub-functions of EF can be improved *via* increasing the functional network connection between these brain regions. In addition, exercise can also improve EF by enhancing neural information transmission (Harrison et al., 2005; Voss et al., 2016; Erickson et al., 2019). The study of Venkareaman et al. further confirmed that compared with the control group, the 12-month walking intervention significantly improved the accuracy of Digit symbol substitution tasks in the elderly people, and was positively correlated with the activation of the left middle frontal gyrus and the right parietal cortex (Venkatraman et al., 2010). A randomized controlled study of patients with MCI showed that aerobic exercise showed a wide range of cognitive improvements compared with the control group. Aerobic fitness was particularly correlated with the larger thickness of (DlPFC) in the dorsolateral prefrontal cortex, while the volume of hippocampus was positively correlated with aerobic fitness over time, indicating that the exercise dose was related to the increase of brain-derived neurotrophic factor, as well as the increase of gray matter volume in PFC and ACC. The study also pointed out that the improvement of memory is related to the increase of DLPFC, and the improvement of EF is related to the increase of miRNA-9 expression (Anderson-Hanley et al., 2018).

Future research is bound to continue to start from the perspective of measurement methods, adding more measurements such as neurophysiology and imaging indicators, to further explore the physiological mechanism of exercise intervention to improve EF.

## Conclusion

The results of this system review support the view that exercise intervention is beneficial to improve the executive function of the middle-aged and elderly people. In terms of exercise prescription, we found that the intervention with a single intervention duration of 30–60 min, at least twice a week, and a total duration of more than 12 weeks showed a good intervention effect. However, the interpretation of the results should be cautious, as there is still some limitations in the included studies. The sample size of some of the included studies is small. The duration and frequency of intervention are short, the mode of intervention is single, and the standard of intervention intensity is vague.

In terms of mechanism, exercise intervention can induce individual cognitive reserve, maintain the activation of EF network in prefrontal cingulate cortex, reduce the loss of brain tissue during aging, and protect the function of specific brain areas. At the same time, exercise intervention can also affect the synthesis of neurotransmitters to a certain extent, to regulate the expression level of related target genes and the excitability of hippocampal synapses, and then delay the decline of cognitive function in the elderly people.

## Data availability statement

The original contributions presented in the study are included in the article/supplementary material, further inquiries can be directed to the corresponding author/s.

## Author contributions

CX generated the idea, conducted literature search, and drafted the paper. JZ and XS conducted literature search and revised the paper. All authors contributed to the article and approved the submitted version.

## Funding

This study was supported by the National Social Science Fund of China (NO. 21BTY095). The funders had no role in study design, data collection and analysis, decision to publish, or preparation of the manuscript.

## Conflict of interest

The authors declare that the research was conducted in the absence of any commercial or financial relationships that could be construed as a potential conflict of interest.

## Publisher's note

All claims expressed in this article are solely those of the authors and do not necessarily represent those of their affiliated organizations, or those of the publisher, the editors and the reviewers. Any product that may be evaluated in this article, or claim that may be made by its manufacturer, is not guaranteed or endorsed by the publisher.
